# High-intensity double-pulse X-ray free-electron laser

**DOI:** 10.1038/ncomms7369

**Published:** 2015-03-06

**Authors:** A. Marinelli, D. Ratner, A. A. Lutman, J. Turner, J. Welch, F.-J. Decker, H. Loos, C. Behrens, S. Gilevich, A. A. Miahnahri, S. Vetter, T.J. Maxwell, Y. Ding, R. Coffee, S. Wakatsuki, Z. Huang

**Affiliations:** 1 SLAC National Accelerator Laboratory, 2575 Sand Hill Road, Menlo Park, California 94025, USA; 2 Deutsches Elektronen-Synchrotron DESY, Notkestrasse 85, Hamburg 22607, Germany; 3 Department of Structural Biology, School of Medicine, Stanford University, Stanford, California 94305, USA

## Abstract

The X-ray free-electron laser has opened a new era for photon science, improving the X-ray brightness by ten orders of magnitude over previously available sources. Similar to an optical laser, the spectral and temporal structure of the radiation pulses can be tailored to the specific needs of many experiments by accurately manipulating the lasing medium, that is, the electron beam. Here we report the generation of mJ-level two-colour hard X-ray pulses of few femtoseconds duration with an XFEL driven by twin electron bunches at the Linac Coherent Light Source. This performance represents an improvement of over an order of magnitude in peak power over state-of-the-art two-colour XFELs. The unprecedented intensity and temporal coherence of this new two-colour X-ray free-electron laser enable an entirely new set of scientific applications, ranging from X-ray pump/X-ray probe experiments to the imaging of complex biological samples with multiple wavelength anomalous dispersion.

## Abstract (web summary)

Two-colour X-ray pulses from free-electron lasers can be used to probe ultrafast dynamics, but the total power is a fraction of the saturation power. Here, Marinelli *et al*. use twin electron bunches to reach full saturation power and increase the two-colour intensity by an order of magnitude at hard-X-ray energies.

## Graphical abstract

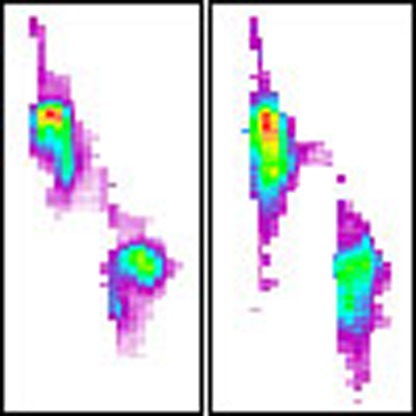

The X-ray free-electron laser (XFEL) is the brightest source of X-rays for scientific applications^1^,^2^,^3^,^4^. The unique properties of XFELs have attracted the interest of a wide community of scientists (for example, see refs 5, 6, 7). Despite the enormous success of XFELs, the effort to improve and extend their capabilities is growing steadily, fueled by user demands for new modes of operation^4^,^8^,^9^,^10^,^11^,^12^ and more precise photon and electron diagnostics^13^,^14^,^15^.

Two-colour pulses are an example of custom-made X-rays from a free-electron laser (FEL), where two pulses of different photon energy and with a variable time delay are generated. Two-colour X-rays have received considerable attention at many FEL facilities worldwide^16^,^17^,^18^,^19^,^20^,^21^. This mode of operation allows users to probe the dynamics of ultra-fast processes triggered by a high-intensity X-ray pump, with a time resolution on the order of a few femtoseconds. For example, in the field of time-resolved resonant X-ray spectroscopy, two colour pulses allow the selective excitation of molecular and atomic processes, such as chemical bond breakage and rearrangement. High-intensity two-colour FELs also allow the study of warm dense matter with time-resolved X-ray pump/X-ray probe experiments^22^,^23^ as well as the experimental investigation of X-ray-induced Coulomb explosion in atom clusters and nanocrystals at the femtosecond scale. Finally, in the field of coherent X-ray imaging, there is a widespread interest in extending multiple wavelength anomalous dispersion (MAD) imaging^24^ to fourth-generation light sources using serial femtosecond crystallography^5^.

In an XFEL, an intense electron bunch travels in a magnetic undulator, generating a high-power X-ray pulse (ranging form a few GW to several tens of GW) with narrow bandwidth (between 0.005 and 0.1%) and duration between a few femtoseconds and a few hundred femtoseconds^1^. The central wavelength *λ*_r_ is given by the resonance formula^25^


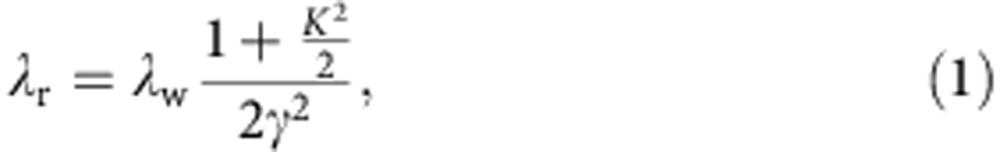


where *λ*_w_ is the undulator period, *γ* is the beam’s Lorentz factor and *K* the scaled amplitude of the magnetic field. At X-ray energies, the methods developed so far rely on generating two X-ray colours by using two distinct values of 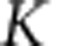 with a quasi mono-energetic electron beam^16^,^20^,^21^. Although this approach can achieve full control of the time and energy separation, the intensity of both pulses is lower than the saturation level because the same electron bunch is used for lasing twice, yielding a total power typically between 5 and 15% of the full saturation power.

Here we show how two independent electron bunches of different energies can be used to generate two X-ray pulses in one undulator (we will refer to this technique as the twin-bunch method). Our method builds on the recent application of pulse-stacking techniques to high-brightness electron injectors^19^,^26^,^27^. In this case, each X-ray pulse is generated by one electron bunch and can reach the full saturation power, improving the two-colour intensity by one order of magnitude at hard X-ray energies. In addition to improving the peak power of two-colour FELs, twin-bunches allow the use of MAD imaging techniques at XFEL facilities by combining two-colour FELs with the existing hard X-ray self-seeding capability^8^. This new capability has been successfully tested in user experiments at Linac Coherent Light Source (LCLS) in a wide variety of fields.

## Results

### Twin-bunch experiment at hard X-rays

**Figure 1:**
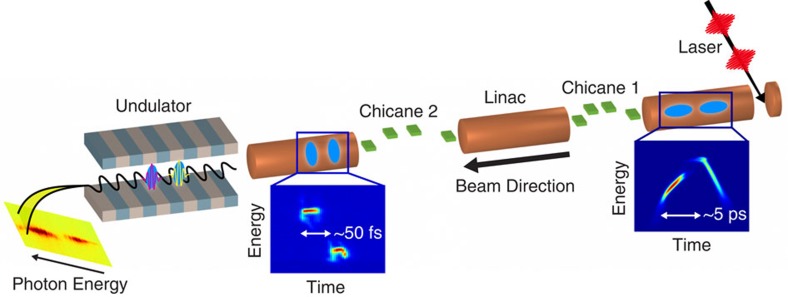
Schematic representation of the experiment. Illustration not to scale. From right to left: a laser pulse train generates two electron bunches at a photocathode (the right inset shows the measured longitudinal phase-space at the photo-injector exit). The two bunches are accelerated in the LCLS linac and compressed by means of two magnetic chicanes (the left inset shows the measured phase-space at the end of the beam line). Finally, the two bunches are sent to an undulator for the emission of two X-ray FEL pulses. The two X-ray pulses have a tunable energy difference in the range of a few percent and a variable time delay of tens of fs.

The twin-bunch method is schematically illustrated in Fig. 1. The electrons are generated by a photocathode illuminated by a train of two laser pulses (generated with a pulse stacker, see the Methods section) with a variable delay on the order of a few picoseconds, generating two separate electron bunches. The two bunches are accelerated up to 15 GeV in the LCLS linear accelerator and compressed from a peak current of 20 A to roughly 4 kA by means of two magnetic chicanes. As a result of the bunch compression, the final arrival time difference of the electron bunches is on the order of a few tens of femtoseconds. As the acceleration/compression system generates a time–energy correlation in the electron beam, the two bunches also have different energies at the end of the accelerator. Finally, the two compressed bunches are sent into the undulator where they emit two X-ray pulses of different energies. Although we will present experimental data at a photon energy of 8.3 keV, the scheme described can work at any photon energy in the available LCLS range (nominally from 300 to 10 keV).

**Figure 2:**
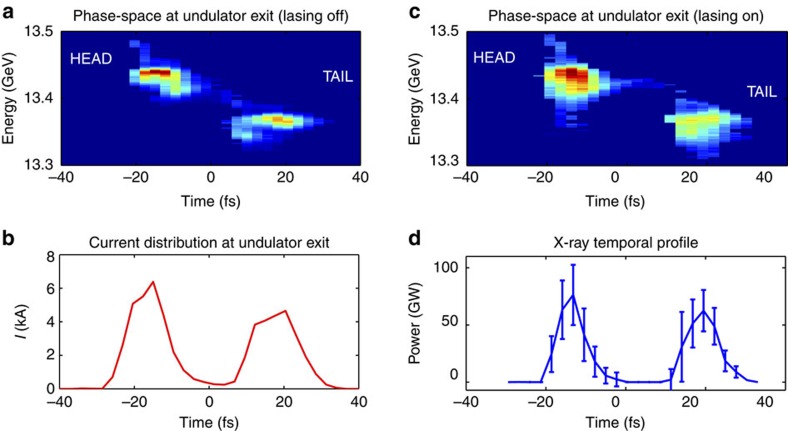
Time-resolved measurements. (**a**) Measured longitudinal phase-space of the two unspoiled electron bunches at the end of the beam-line (the FEL process being suppressed). (**b**) Associated current profile of the two bunches. (**c**) Measured longitudinal phase-space of the two bunches after lasing. (**d**) Temporal profile of the two X-ray pulses reconstructed from the two phase-space measurements. The error bars are derived from the averaging of 100 unspoiled phase-space measurements, as discussed in ref. 13. The horizontal axis represents the arrival time with respect to a fixed observer (the beam head is on the left).

The recently developed X-band transverse deflector^13^ provides an effective diagnostic tool for this two-bunch technique. Figures 2a and 2c show the measured longitudinal phase-space of the two bunches at the end of the undulator beamline for the unspoiled beam (that is, suppressing the lasing process with a large transverse perturbation in the electron orbit) and for the beam after the lasing process. The peak current is roughly 5 kA for a total charge of 150 pC, with an energy separation of 70 MeV. Figure 2d shows the temporal profile of the X-ray pulses reconstructed from the longitudinal phase-space measurement (see the Methods section and ref. 13). The two bunches emit two X-ray pulses with a peak power of 76 GW for the head pulse and 62 GW for the tail, and a full-width at half-maximum (FWHM) pulse duration of approximately 8 and 10 fs, respectively. The two pulses are not Fourier transform limited as the FEL process is initiated by noise in the electron distribution, a mode of operation commonly referred to as self-amplified spontaneous emission or SASE^28^. The fine spiky temporal structure of the two SASE pulses is not resolved by this diagnostic, which at this energy has a time resolution of 4 fs root mean square (RMS). For this data set, the pulse energy averaged over 100 shots is 1.207 mJ with a shot-to-shot fluctuation level of 12%. These data illustrate the main advantage of this two-colour scheme, which is that of generating peak power levels and pulse energies comparable to the standard SASE operation of LCLS, improving the performance by over an order of magnitude compared with other two-colour methods at LCLS and other user facilities^16^,^20^,^21^ (we note that although the results published on single-bunch two-colour techniques at LCLS report measurements performed at soft X-ray energies, unpublished measurements at hard X-rays yield a pulse energy on the order of 100 μJ for double the pulse duration reported here).

### Spectral properties

**Figure 3:**
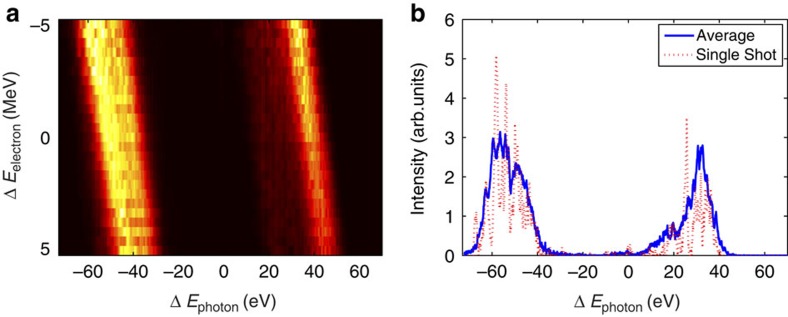
Spectral measurements: SASE. (**a**) Spectral intensity as a function of beam energy and photon energy in the SASE regime. (**b**) Average and single-shot spectrum for a fixed beam energy (the data are binned over electron beam energy fluctuations).

To illustrate the double-colour structure of the X-rays, Fig. 3 shows X-ray spectra taken under the same beam conditions as Fig. 2. Figure 3a shows the spectral intensity as a function of electron-beam energy and photon energy. The data are binned in beam energy using a single-shot measurement of the average electron energy in order to deconvolve the effect of shot-to-shot energy fluctuations. The peak-to-peak energy separation of the two colours is 90 eV centred around 8.3 keV. The photon energy of the two pulses is correlated to the beam energy and the colour separation is independent of the average beam energy variations (note that this implies that the fluctuations of the energies of the two bunches are correlated). The bandwidth of the two pulses differs slightly. This is because of different features in the longitudinal phase-space of the two bunches. Although this effect could, in principle, be controlled by varying the strength of the LCLS linearizing RF cavity, the typical tuning procedure is focused on balancing the peak power and time duration of the two X-ray pulses. Figure 3b shows a single-shot spectrum and an average spectrum for a fixed beam energy.

The energy separation of the two colours can be tuned independently of the other main beam parameters (such as peak current and time arrival separation) by varying the amount of compression in the first magnetic chicane (see the Methods section for further details). Separations as high as 120 eV have been generated during delivery to user experiments at this average photon energy. Although the spectral separation in this experiment is limited by the photon diagnostic, we estimate that the maximum separation achievable is limited to a few percent by chromatic effects in the electron beam transport system. Specific chromaticity correction methods could be adopted to increase the energy acceptance of the transport system, and hence the achievable colour separation.

**Figure 4:**
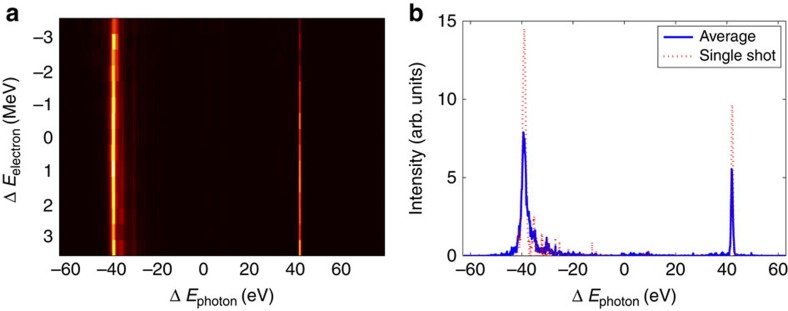
Spectral measurements: self-seeding. (**a**) Spectral intensity as a function of beam energy and photon energy in the self-seeded regime. (**b**) Average and single-shot spectrum for a fixed beam energy (the data are binned over electron beam energy fluctuations).

It has been recently demonstrated that the LCLS self-seeding system can be tuned to select two narrow spectral lines^29^. This is accomplished by means of two independent rotations of the self-seeding diamond crystal. The two degrees of freedom are used to select two nearby crystal reflections, causing two distinct photon energies to be present in the forward transmitted seeding X-ray pulse. The double bunch mode presented here has been combined with the two-colour self-seeding scheme to obtain two widely spaced narrow X-ray spectra. Figure 4 shows the spectral intensity as a function of beam and photon energy (a) and single-energy spectra for both averaged and single-shot (b). As the crystal selects two photon energies regardless of the beam energy, the two colours appear as two vertical lines in the binned data (as opposed to Fig. 3a). In the self-seeded mode, the average pulse energy is 130 μJ with 50% shot-to-shot intensity fluctuations. The spectral intensity of the two colours is improved by a factor 2 compared with the SASE mode and the bandwidth decreases to 1.6 and 0.8 eV for the low- and high-energy colours, respectively. The difference in bandwidth between the two colours is likely due to a difference in the second-order time–energy correlation of the two electron bunches^30^.

### Time delay tunability

The ability to vary the time delay between two pulses without affecting their energy separation or their individual pulse duration is a crucial feature for X-ray pump/X-ray probe experiments. This goal is achieved in the two-bunch mode thanks to the two-stage bunch compression system.

**Figure 5:**
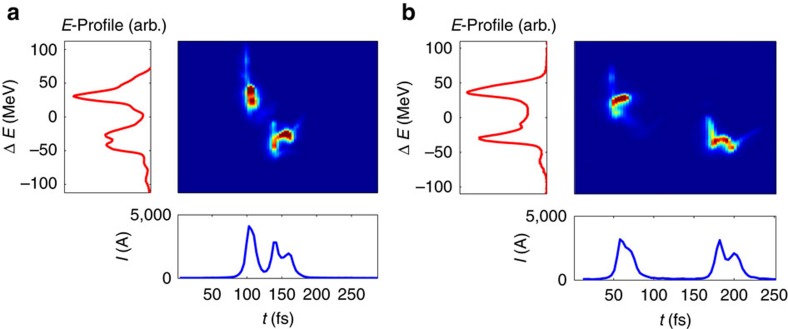
Final time delay variation. Phase-space, current profile and energy profile of the two bunches for an initial delay of 8 ps (**a**) and 12 ps (**b**). The final time delays are 30 and 125 fs. Both the energy separation and the peak current are unchanged.

Figure 5 shows the measured phase space, as well as the projected current profile and the energy profile of the two bunches for two configurations yielding an arrival time delay of 30 and 125 fs. The energy difference is unchanged in the two cases, as well as the peak current (except for a variation in the head bunch, which is within the typical fluctuation level). The two sets of data were measured using two different initial time delays at the cathode (8 and 12 ps, respectively) and by keeping the energy separation and final peak current constant with the use of feedback systems acting on the two compression chicanes. The time delay jitter depends on the specific tune of the machine, however, it generally varies between 3 and 7 fs and it is induced by the phase-fluctuations of the accelerating field before the second bunch compressor.

We note that the range of tunability is dependent on the chosen peak current, which in turn is constrained by the energy of operation. For peak currents in ranging between 3 and 5 kA, which are normally used at hard X-rays, the high-energy pulse typically arrives first, with a delay ranging from 0 to roughly 100 fs. For lower peak currents (that is, at soft X-rays), the arrival time can be inverted. The arrival time of the two pulses at hard X-rays could be inverted with the construction of a dedicated magnetic chicane before the undulator entrance, an option that is currently being considered for LCLS.

## Discussion

The double-electron bunch method demonstrated here allows the generation of mJ-level two-colour X-rays with tunable energy separation and time delay. This performance improves the intensity of two-colour XFELs by over an order of magnitude with respect to the current state of the art. In addition to the larger peak power, the double-bunch mode presents several other advantages over standard single-bunch methods. First, the two X-ray pulses have the same source point, improving their focusing properties at the down-stream experimental stations. Second, the radiation pulses can be diagnosed independently and non-destructively using longitudinal phase-space diagnostics on the dumped electrons (unlike single-bunch methods, where this diagnostic would only reconstruct the projection of both pulses). Finally, as both colours are amplified in the entire undulator length, this scheme can be coupled to a two-colour self-seeding system to yield two nearly Fourier transform limited pulses. The energy difference, duration and arrival time of the two X-ray pulses can be varied independently of each other. This feature makes this scheme an ideal candidate for time-resolved X-ray pump/X-ray probe experiments.

These results have a direct impact on the broad field of photon science by extending the scientific capabilities of fourth-generation light sources. Of particular importance, is the generation of two widely spaced seeded pulses, which allows the collection of two simultaneous diffraction patterns at different X-ray energies on a single detector. This enables the use of MAD techniques for the experimental phase determination of X-ray diffraction data at XFEL facilities.

This new XFEL capability has received considerable attention from the XFEL user community and it has already been successfully delivered to several user experiments in a wide range of photon energies. In addition to enabling new science with XFELs, this experiment demonstrates, for the first time, the generation, compression and temporal shaping of double-electron bunches from a photo-injector-driven linac in the tens of GeV range, with applications in the rapidly growing fields of plasma and dielectric wakefield accelerators^31^,^32^,^33^,^34^.

## Methods

### Instrumentation

The LCLS undulator is composed of 31 sections with a period *λ*_w_=3 cm, each section is 3.4 m long. The undulator parameter is *K*=3.5 with a tunability of roughly ±1%.

The two cathode laser pulses are generated with a pulse stacker. The stacker is composed of a polarizer, which splits the two polarizations into different paths, and a motorized delay stage that varies the time delay between the two laser pulses. The relative intensity of the two pulses is controlled by means of a wave-plate and is adjusted to yield the same charge for both bunches (note that typically this requires different pulse energy on the two arms because of the different Schottky phase). Each laser pulse has a Gaussian-like temporal shape with a duration of 2.5 ps FWHM. Owing to space-charge effects, each electron bunch is lengthened to roughly 3.2 ps FWHM in the injector.

The electron longitudinal phase-space is measured with an X-band deflecting radio-frequency cavity (XTCAV)^13^. The X-ray temporal profile is reconstructed from the time-resolved measurement of the energy transfer from the electrons to the radiation field obtained with the XTCAV, as described in ref. 13. The spectra are measured by means of a bent crystal spectrometer with a full spectral range of about 120 eV (ref. 35).

### Beam parameters tuning

The tuning of the twin-bunch system involves three global constraints: setting an energy difference, setting a certain peak current (which in turn determines the FEL power gain and the pulse duration) and setting a certain time delay. These three constraints can be satisfied by using three independent variables in the beam transport: bunch compression in the first and second chicane, and initial pulse delay at the cathode. Although these three variables are not independent of each other, each one of them has a strong effect on one of the three parameters. The compression in the first chicane controls the final energy difference (since most of the energy-chirp in the linac is introduced between the first and second chicanes), the final peak current is controlled by the second bunch compression stage. The remaining parameter is the initial cathode delay and it can be used to vary the final time delay provided that the energy difference and peak current are compensated for using the other two variables. This is usually accomplished with a peak current feedback system.

We note that, with respect to the standard beam transport configuration, the only significant difference consists of a lower compression factor after the first bunch compressor by roughly a factor 2 or more, depending on the desired energy separation. This ensures that the two electron bunches are not overlapped in time in the linac between the two magnetic chicanes. This serves two main purposes: (i) preserving the linearity of the bunch compression process (since a partial time-overlap before the second compression stage would generate strongly non-linear wake fields); (ii) generating a significant energy difference between the two bunches (since the energy separation is almost entirely generated by the off-crest accelerating field between the two bunch compressors). A detailed description of the beam dynamics of twin bunches will be the subject of a supporting publication.

## Author contributions

A.M., D.R., A.A.L., R.C., S.W. and Z.H. designed the experiment. A.M., D.R., R.C., S.W. and Z.H. co-wrote the paper with input from all the co-authors. All co-authors contributed to the realization of the experiment. A.M., T.J.M., A.A.L., C.B. and Y.D. performed the data analysis.

## Additional information

**How to cite this article:** Marinelli, A. *et al*. High-intensity double-pulse X-ray free-electron laser. *Nat. Commun*, 6:6369 doi: 10.1038/ncomms7369 (2015).

## References

[b1] Emma P. . First lasing and operation of an angstrom-wavelength free-electron laser. Nat. Photon. 4, 641–647 (2010) .

[b2] Ishikawa T. . A compact X-ray free-electron laser emitting in the sub-angstrom region. Nat. Photon. 6, 540–544 (2012) .

[b3] Ackermann W. . Operation of a free-electron laser from the extreme ultraviolet to the water window. Nat. Photon. 1, 336–342 (2007) .

[b4] Allaria E. . Two-stage seeded soft-X-ray free-electron laser. Nat. Photon. 7, 913–918 (2013) .

[b5] Chapman H. N. . Femtosecond X-ray protein nanocrystallography. Nature 470, 73–77 (2011) . 21293373 10.1038/nature09750 PMC3429598

[b6] Rohringer N. . Atomic inner-shell X-ray laser at 1.46 nanometres pumped by an X-ray free-electron laser. Nature 481, 488–491 (2012) . 22281598 10.1038/nature10721

[b7] Vinko S. M. . Creation and diagnosis of a solid-density plasma with an X-ray free-electron laser. Nature 482, 59–62 (2012) . 22278059 10.1038/nature10746

[b8] Amann J. . Demonstration of self-seeding in a hard-X-ray free-electron laser. Nat. Photon. 6, 693–698 (2012) .

[b9] Allaria E. . Highly coherent and stable pulses from the FERMI seeded free-electron laser in the extreme ultraviolet. Nat. Photon. 6, 699–704 (2012) .

[b10] Wu J., Marinelli A. & Pellegrini C. Generation of longitudinally coherent ultra-high power X-ray pulses by phase and amplitude mixing. Proc. FEL 2012, Nara, Japan 237–240 (2012) .

[b11] McNeil B. W. J., Thompson N. R. & Dunning D. J. Transform-limited X-ray pulse generation from a high-brightness self-amplified spontaneous-emission free-electron laser. Phys. Rev. Lett. 110, 134802 (2013) . 23581327 10.1103/PhysRevLett.110.134802

[b12] Xiang D., Ding Y., Huang Z. & Deng H. Purified self-amplified spontaneous emission free-electron lasers with slippage-boosted filtering. Phys. Rev. Spec. Top. Accel. Beams 16, 010703 (2013) .

[b13] Behrens C. . Few-femtosecond time-resolved measurements of X-ray free-electron lasers. Nat. Commun. 5, 3762 (2014) . 24781868 10.1038/ncomms4762

[b14] Harmand M. . Achieving few-femtosecond time-sorting at hard X-ray free-electron lasers. Nat. Photon. 7, 215–218 (2013) .

[b15] Hartmann N. . Sub-femtosecond precision measurement of relative X-ray arrival time for free-electron lasers. Nat. Photon. 8, 706–709 (2014) .

[b16] Lutman A. A. . Experimental demonstration of femtosecond two-color x-ray free-electron lasers. Phys. Rev. Lett. 110, 134801 (2013) . 23581326 10.1103/PhysRevLett.110.134801

[b17] De Ninno G. . Chirped seeded free-electron lasers: self-standing light sources for two-color pump-probe experiments. Phys. Rev. Lett. 110, 064801 (2013) . 23432255 10.1103/PhysRevLett.110.064801

[b18] Allaria E. . Two-colour pump-probe experiments with a twin-pulse-seed extreme ultraviolet free-electron laser. Nat. Commun. 4, 2476 (2013) . 24048228 10.1038/ncomms3476 PMC3791458

[b19] Petrillo V. . Observation of time-domain modulation of free-electron-laser pulses by multipeaked electron-energy spectrum. Phys. Rev. Lett. 111, 114802 (2013) . 24074094 10.1103/PhysRevLett.111.114802

[b20] Hara T. . Two-colour hard X-ray free-electron laser with wide tunability. Nat. Commun. 4, 2919 (2013) . 24301682 10.1038/ncomms3919

[b21] Marinelli A. . Multicolor operation and spectral control in a gain-modulated x-ray free-electron laser. Phys. Rev. Lett. 111, 134801 (2013) . 24116783 10.1103/PhysRevLett.111.134801

[b22] Lee R. W. . Plasma-based studies with intense X-ray and particle beam sources. Laser Particle Beams 20, 527 (2002) .

[b23] Forsman A., Ng A., Chiu G. & More R. Interaction of femtosecond laser pulses with ultrathin foils. Phys. Rev. E 58, R1248 (1998) .

[b24] Hendrickson W. A. & Ogata C. M. Phase determination from multiwavelength anomalous diffraction measurements. Methods Enzymol. 276, 494 (1997) . 10.1016/S0076-6879(97)76074-9 27799111

[b25] Bonifacio R., Pellegrini C. & Narducci L. Collective instabilities and high-gain regime in a free electron laser. Opt. Commun. 50, 373 (1984) .

[b26] Musumeci P., Li R. K. & Marinelli A. Nonlinear longitudinal space charge oscillations in relativistic electron beams. Phys. Rev. Lett. 106, 184801 (2011) . 21635094 10.1103/PhysRevLett.106.184801

[b27] Ferrario M. . Laser comb with velocity bunching: preliminary results at SPARC. Nucl. Instrum. Meth. Phys. Rev. A 637, S43–S46 (2011) .

[b28] Bonifacio R., De Salvo L., Pierini P., Piovella N. & Pellegrini C. Spectrum, temporal structure, and fluctuations in a high-gain free-electron laser starting from noise. Phys. Rev. Lett. 73, 70 (1994) . 10056722 10.1103/PhysRevLett.73.70

[b29] Lutman A. A. . Demonstration of single-crystal self-seeded two-color x-ray free-electron lasers. Phys. Rev. Lett. 113, 254801 (2014) . 25554887 10.1103/PhysRevLett.113.254801

[b30] Marinelli A. . Comparative study of nonideal beam effects in high gain harmonic generation and self-seeded free electron lasers. Phys. Rev. Spec. Top. Accel. Beams 13, 070701 (2010) .

[b31] Rosenzweig J. B. . Experimental observation of plasma wake-field acceleration. Phys. Rev. Lett. 61, 98 (1988) . 10038703 10.1103/PhysRevLett.61.98

[b32] Blumenfeld I. . Energy doubling of 42 GeV electrons in a metre-scale plasma wakefield accelerator. Nature 445, 741–744 (2007) . 17301787 10.1038/nature05538

[b33] Andonian G. . Dielectric wakefield acceleration of a relativistic electron beam in a slab-symmetric dielectric lined waveguide. Phys. Rev. Lett. 108, 244801 (2012) . 23004279 10.1103/PhysRevLett.108.244801

[b34] Litos M. . High-efficiency acceleration of an electron beam in a plasma wakefield accelerator. Nature 515, 92–95 (2014) . 25373678 10.1038/nature13882

[b35] Zhu D. . A single-shot transmissive spectrometer for hard x-ray free electron lasers. Appl. Phys. Lett. 101, 034103 (2012) .

